# Efficiency of anchorage systems for RC beams strengthened in flexure using basalt fiber reinforced polymers

**DOI:** 10.1038/s41598-026-52540-5

**Published:** 2026-05-26

**Authors:** John Aziz, Mohamed Ragab, Fareed Elgabbas, Ibrahim Abdel-Latif

**Affiliations:** Structural Department, Faculty of Engineering, Ain-Shams University, 1 El-Sarayat Street, Abbasya, Cairo, 11535 Egypt

**Keywords:** Basalt, Fiber reinforced polymer (FRP), Anchorage, Flexure, Strengthening, Deflection, Debonding, U-wraps, Spike, Ductility, Engineering, Materials science

## Abstract

Recently, Basalt Fiber Reinforced Polymer (BFRP) composites emerged as a new FRP type, in addition to the commonly used glass, carbon, and aramid. The common premature debonding failure of externally bonded fiber-reinforced polymer (FRP) composites, when applied to reinforced concrete (RC) structures, has made searching for efficient anchorage systems an inevitable and challenging issue. Many studies through experimental testing and numerical modeling verified that anchorages applied to FRP systems not only enhance the member’s ductility and strength but also prevent the typical debonding of the FRP at a low strain level compared to the rupture strain. Research is needed, however, to understand the efficiency of different anchorage systems when applied to relatively high-strain BFRP sheets to strengthen concrete members. This research presents an experimental study aimed at investigating the efficiency of using anchorage systems in enhancing the flexural behavior of concrete beams strengthened with BFRP sheets. A total of eight concrete beams measuring 3100 mm length, 150 mm width and 350 mm depth were constructed and tested up to failure. The test parameters were the number of BFRP layers, the development length, and anchorage systems. The beam specimens were designed in accordance with ACI 440.2R-17 and tested under four-point bending over a clear span of 2800 mm until failure. The results showed that BFRP strengthening enhanced the flexural capacity of beams by up to 33% compared to the control specimen. However, increasing the number of BFRP layers without proper anchorage did not significantly improve strength due to premature debonding. The use of U-wrap anchorage successfully changed the failure mode from debonding to BFRP rupture, leading to more efficient utilization of the composite material, with anchorage effectiveness factor *k*_*fab*_ = 2.36, while spike anchors with anchor dowels 150 mm inside the concrete have an anchorage effectiveness factor *k*_*fab*_ = 1.97 which showed limited effectiveness depending on embedment depth. In addition, strengthened beams exhibited a reduction in ductility of approximately 28% compared to the control beam. The findings highlight the critical role of anchorage systems in achieving optimal performance of BFRP-strengthened RC beams.

## Introduction

Structural deficiency and degradation of concrete structures due to aging, excessive load, or seismic considerations necessitate the need for cost-effective and sustainable solutions. Conventional techniques for strengthening reinforced concrete (RC) structures such as using bonded steel plates, steel or concrete jackets, and external prestressing techniques often entail extensive construction processes, which cause disruption and inconvenience. Introducing fiber-reinforced polymers (FRPs) has brought about a paradigm shift in strengthening concrete structures techniques. Carbon, glass, and aramid fibers have demonstrated their effectiveness in enhancing the load-bearing capacity due to their high strength-to-weight ratio, thermal resistance, and ease of installation^1,2^. However, Basalt Fiber-Reinforced Polymer (Basalt FRP), derived from naturally occurring basalt rock, is gaining prominence due to its advantageous properties compared to other fiber-reinforced polymers^3,4^. For example, BFRP has higher strength and modulus of elasticity, similar cost, and more chemical stability than E-glass FRP^5^. Moreover, it is significantly less expensive than CFRP and can operate at a larger temperature range^6,7^.

**Fig. 1:**
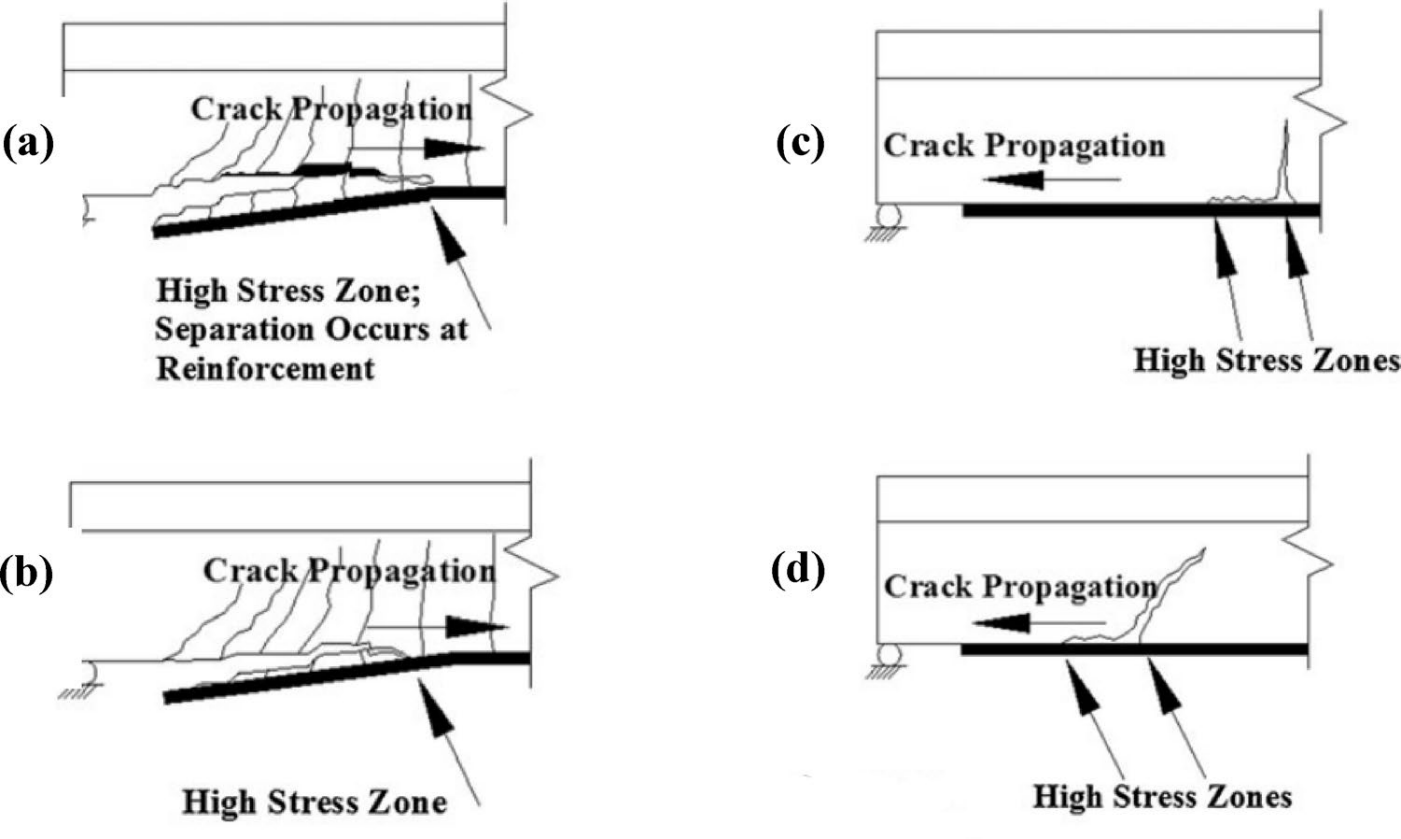
Debonding modes of failure^1^.

Unfortunately, the premature debonding of the FRP from the concrete substrate, which is a sudden and brittle failure, usually overrides the improved material properties of FRPs. Therefore, it is critical to design against the several debonding failure modes: (a) Concrete cover separation failure^8^; (b) Plate end interfacial debonding^9^; (c) Intermediate flexural crack-induced interfacial debonding (Otherwise known as IC debonding)^10^; and (d) Intermediate flexural shear crack-induced interfacial debonding^11,12^. The typical debonding failure modes indicated in Fig. 1 make achieving failure through FRP rupture a difficult challenge.

Debonding modes of failure are undesired because they usually happen well before the section’s predicted ultimate strength is reached, which causes the FRP to be underutilized and reduces the ductility of the strengthened member^13,14^. Augmentation of anchorage systems helps transfer the failure mode of the strengthened element from brittle to more ductile as well as enhancing the safety and reliability of the structure^15^.

Recent research has increasingly focused on enhancing the structural performance of reinforced concrete (RC) members through advanced strengthening techniques that go beyond conventional externally bonded FRP systems. For instance, externally applied post-tensioning systems have been shown to significantly improve the shear behavior and load-carrying capacity of RC beams by actively controlling crack development and redistributing internal stresses^16^. Similarly, near-surface mounted (NSM) reinforcement techniques, including the use of aluminum or composite sections, have demonstrated improved bond performance and enhanced shear ductility due to better stress transfer mechanisms between the strengthening material and the concrete substrate. These approaches highlight the importance of not only increasing strength but also improving deformation capacity and failure control in strengthened RC elements. Moreover, these findings collectively underline that the effectiveness of any strengthening system is highly dependent on bond characteristics, anchorage efficiency, and crack control mechanisms^17^.

The most used externally bonded (EB) anchorage systems are FRP U-wraps (U-jacket), FRP spike anchors (fan anchors), π-anchors, and steel clamps^18,19^. Many studies are needed to fill the gap of knowledge between design codes and applications of anchorage systems to enable the widespread use of FRP anchorage systems. Presently, the lack of reasonable and reliable design guidelines is the main barrier obstructing the broad implementation of FRP anchorage systems. Consequently, ACI 440.2R-17^20^ design guideline states that the practical application of anchorage systems must be supported by representative experimental testing.

Few studies were conducted to investigate the behavior of RC beams strengthened in flexure using Basalt FRP. Duic et al. (2018)^21^ tested seven reinforced concrete beams (275 × 500 × 3200 mm) strengthened with different numbers of BFRP layers in flexure up to failure. The beams strengthened with three BFRP layers without anchorages failed by premature debonding of BFRP, while BFRP rupture was achieved when U-wraps were added. It was concluded that load-carrying capacities in strengthened beams were enhanced by up to 25% with only three layers of BFRP composite. Moreover, it was found that the ductility of beams strengthened with BFRP layers reduced with a limited reduction of 30%^22^.

Research on U-jacket anchors is substantial and has received a lot of attention due to their ease of construction and their improvement to the bond strength at the concrete-FRP laminate interface^24^. Chen et al. (2017)^23^ used basalt FRP laminates to study the effects of different wrapping schemes and concluded that the inclined U-jacket anchors at 45° to the support direction were more effective and had higher peak loads than the vertical one with the same amount of FRP materials; covering the full span by U-jacket anchors provides slight enhancement compared to partial coverage of U-jackets.

This paper presents an experimental study aimed at investigating the flexural behavior and serviceability performance of RC beams strengthened with BFRP layers. The study also aimed to assess the performance of U-wrapping and FRP spike anchors in enhancing the flexural capacity of concrete beams and changing the mode of failure from debonding to FRP rupture. In addition, the test results were compared to current design provisions and recommendations.

## Experimental program

### Material properties

Material tests were conducted on concrete, steel, and BFRP fabric to ascertain their mechanical properties. The beams were made with normal-strength concrete with a 28-day target compressive strength of 35 MPa. A cubic meter of concrete contained 350 kg of cement, 670 kg of natural sand, 1120 kg of aggregate (10 mm maximum nominal size), 176 L of water, and workability additives of 1.4% of cement weight. Eight beams were cast using two concrete batches. The wet curing process started two hours after the concrete was cast by covering the concrete surface with wet burlap and polythene sheeting for 10 days. Although two concrete batches were used, identical mix proportions and curing conditions were maintained. The measured compressive strengths showed no significant variation. Nevertheless, minor batch-to-batch variations may have slightly influenced cracking and bond-related behavior. The material properties of the steel reinforcement were determined from the uniaxial tension test as specified in ASTM A370-18^24^. The mean yield and ultimate strength of the 10 M longitudinal reinforcement steel bars were 550 and 680 MPa, respectively. The strength and modulus of elasticity of the BFRP fabric were 930 ± 80 MPa and 40 ± 2.4 GPa, respectively, which obtained using coupon tests in accordance with ASTM D3039-17^25^. Table 1 shows the tensile properties of tested BFRP laminates.

**Table 1:** Mechanical properties of BFRP composite.

Property	Value
Fabric weight (g/m^2^)	300
Fabric thickness (mm)	0.17
Ultimate strength, *f*_*u*_ (MPa)	930 ± 80
Ultimate strain, *ε*_*fu*_ (%)	2.3
Modulus of elasticity, *E*_*f*_ (GPa)	40 ± 2.4

### Test specimens and strengthening techniques

**Fig. 2:**
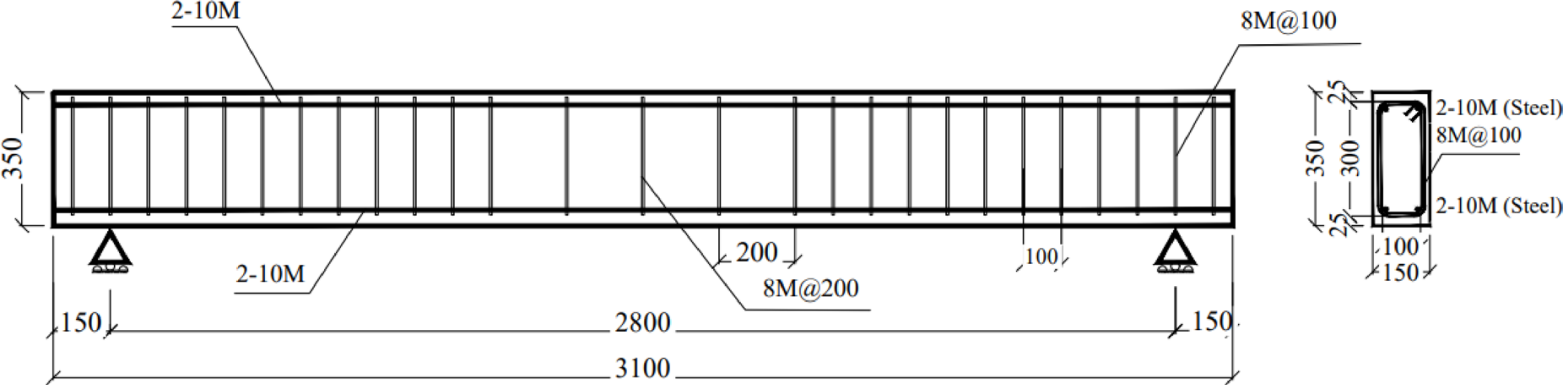
Schematic drawing for the tested beams (in mm).

**Fig. 3:**
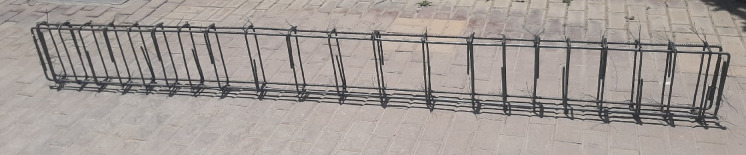
Steel cage for the tested beams.

This study included eight reinforced concrete beams with the same dimensions: width = 150 mm, depth = 350 mm, length = 3100 mm, and span = 2800 mm. All the beams had two 10 M steel bars as top and bottom reinforcement. Shear reinforcement consisting of Ø8 steel stirrups each 100 mm were used in both shear spans to avoid shear failure. To minimize the confining effect of the shear reinforcement on the flexural behavior, Ø8 steel stirrups each 200 mm were used in the constant moment zone. Figure 2 shows the dimensions and reinforcement details of the tested beams, and Fig. 3 shows the actual steel cages for the beams. Tables 2 and 3 describe the tested specimens. The naming convention for the beams consists of the number of the specimen, followed by the length of BFRP layers used in strengthening as a fraction of the span of the beam, followed by the number of BFRP layers, followed by the anchorage technique used. For instance, specimen B6-0.54S-2L-U was beam number 6 strengthened over 54% of beam’s span with 2 layers of BFRP and fixed using U-wraps anchorage system. The first beam B1-C was a control specimen without any layers of BFRP. Specimen B2-S-4L was strengthened on its full span with 4 layers of BFRP passing over the two supports. While specimen B3-S-2L was strengthened by 2 layers of BFRP layers passing over the support and with 2 U-wrap anchors at the beam ends. Specimen B4-0.91S-2L strengthened by 2 layers of BFRP over 91% of beam’s span which is calculated from ACI 440.2R-17^20^ to satisfy the development length required for preventing debonding of FRP layers from concrete substrate. Specimen B5-0.54S-2L strengthened by 2 layers of BFRP over 54% of beam’s span which covers the length of maximum moment between the two concentrated load with additional 350 mm on each side enough for application of anchorage systems.

**Table 2:** Test matrix of specimens.

Beam ID	BFRP layers	Strengthening length (% of span)	Anchorage type	Objective
B1-C	0	0	None	Control specimen
B2-S-4L	4	100	None	Effect of number of layers
B3-S-2L	2	100	U-wraps	Effect of number of layers
B4-0.91S-2L	2	91	None	Development length verification
B5-0.54S-2L	2	54	None	Reduced length without anchorage (new control beam)
B6-0.54S-2L-U	2	54	U-wraps	U-wrap anchorage efficiency
B7-0.54S-2L-SP75	2	54	Spike (75 mm)	Effect of shallow anchors
B8-0.54S-2L-SP150	2	54	Spike (150 mm)	Effect of deeper anchors

**Table 3:** Summary of strengthening configurations and specimen details.

Beam	Description	No. of BFRP Layers	Strengthening Scheme
B1-C	Control beam with no FRP	0	
B2-S-4L	FRP full span and over support	4	
B3-S-2L	FRP full span and over support + U-wraps	2	
B4-0.91S-2L	FRP with curtailment 115 mm from support	2	
B5-0.54S-2L	FRP 1500 mm in the middle	2	
B6-0.54S-2L-U	FRP 1500 mm in the middle with two U-wraps with 100 mm width	2	
B7-0.54S-2L-SP75	FRP 1500 mm in the middle with two spike anchors of 75 mm embedded depth	2	
B8-0.54S-2L-SP150	FRP 1500 mm in the middle with two spike anchors of 150 mm embedded depth	2	

Two different techniques for anchoring the EB BFRP layers were taken into consideration to assess the influence of FRP anchorage on the reinforced beams. The beam identified as B6-0.54S-2L-U had two EB BFRP layers over 54% of beam’s span and anchored by two BFRP U-wrap strips at each end. The width of each U-wrap strip was 100 mm. B7-0.54S-2L-SP75 and B8-0.54S-2L-SP150 were anchored by spike anchors with different depth of anchor dowels embedded inside the concrete. To provide a clear overview of the experimental program, the test matrix of all specimens, including the investigated parameters such as number of BFRP layers, strengthening length, and anchorage type, is summarized in Table 2. Table 3 summarizes with schematic diagrams the different strengthening and anchoring methods implemented. The material characteristics (compressive strength for concrete, yielding strength of steel, modulus of elasticity of steel, number and size of top and bottom rebars) and geometry of the beams (cross-sectional dimensions, span, and test setup) were precisely the same in all the beams.

### Beams preparation for strengthening

**Fig. 4:**
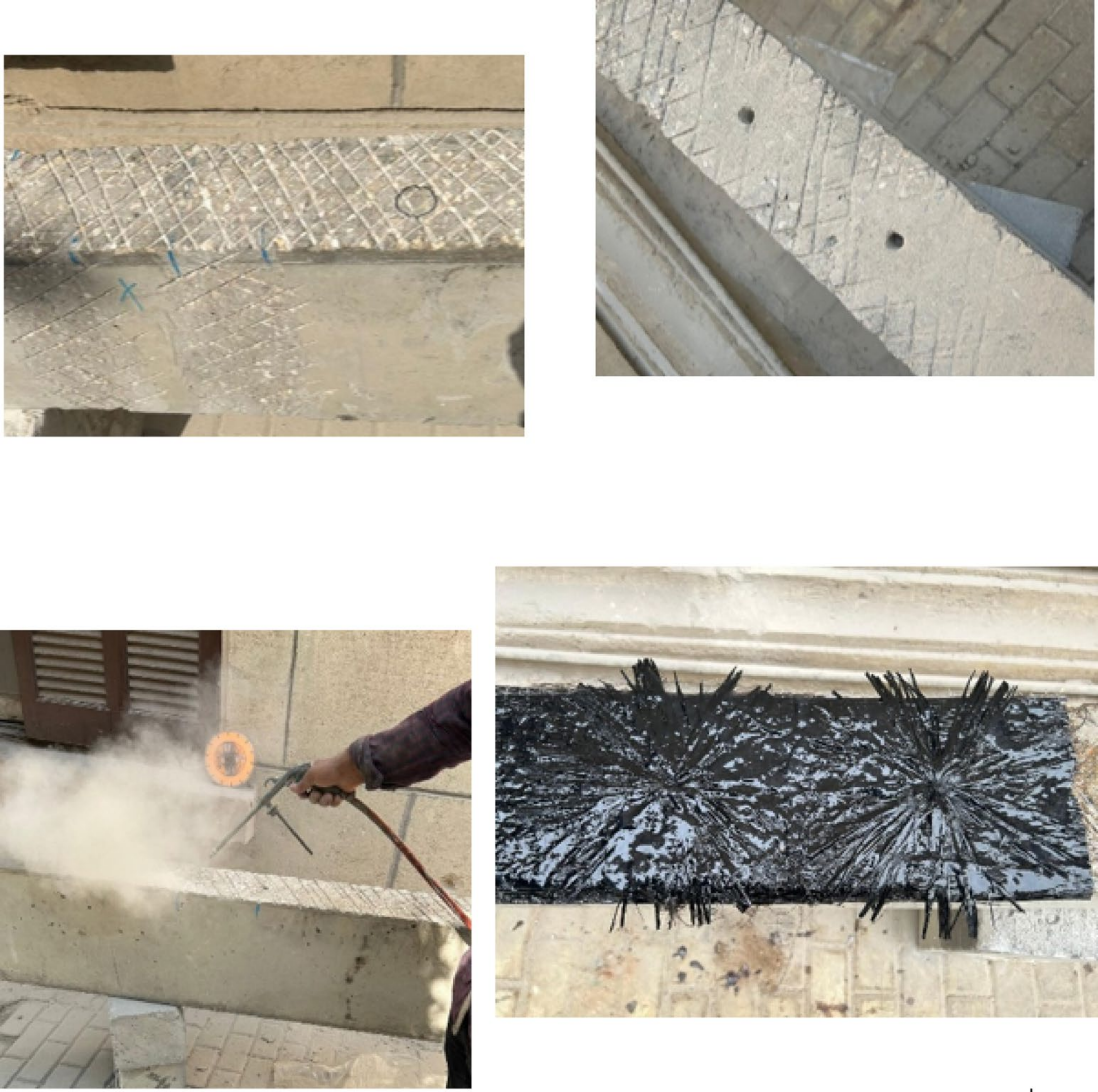
Surface preparations and spike anchors installation.

Prior to adding epoxy resin to fix the BFRP layers, the beams’ surfaces were grinded using steel brushes to improve the adhesion between the concrete and BFRP. The corners of the beams where the U-wrap anchors were to be installed were rounded. In addition, holes and notches with rounded corners were formed in the positions where the FRP spike anchors were to be installed. Following surface preparations, every beam undergoes cleaning and degreasing as shown in Fig. 4.

A polymer-based adhesive system was used to bond the BFRP sheets to the concrete substrate. The system consisted of a two-component polyester-based resin combined with an accelerator, catalyst, and thinner, mixed in accordance with the manufacturer’s recommended proportions. The mixture exhibited a short pot life of approximately 15 min, requiring careful preparation and prompt application.

Based on the data sheet, polyester-based adhesives of this type typically exhibit tensile strengths in the range of 20–40 MPa and elastic moduli between 2 and 4 GPa, with adequate bond strength to mineral substrates when proper surface preparation is ensured. In this study, the concrete surface was mechanically prepared and cleaned prior to application to enhance adhesion. The strengthening system was applied under laboratory ambient conditions, and the specimens were allowed to cure for a minimum of 7 days before testing to ensure sufficient bond development.

It should be noted that the reported tensile properties of the BFRP correspond to the fully cured composite laminate (i.e., fabric impregnated with the adhesive system), as determined from coupon tests conducted in accordance with ASTM D3039.

### Instrumentation

**Fig. 5:**
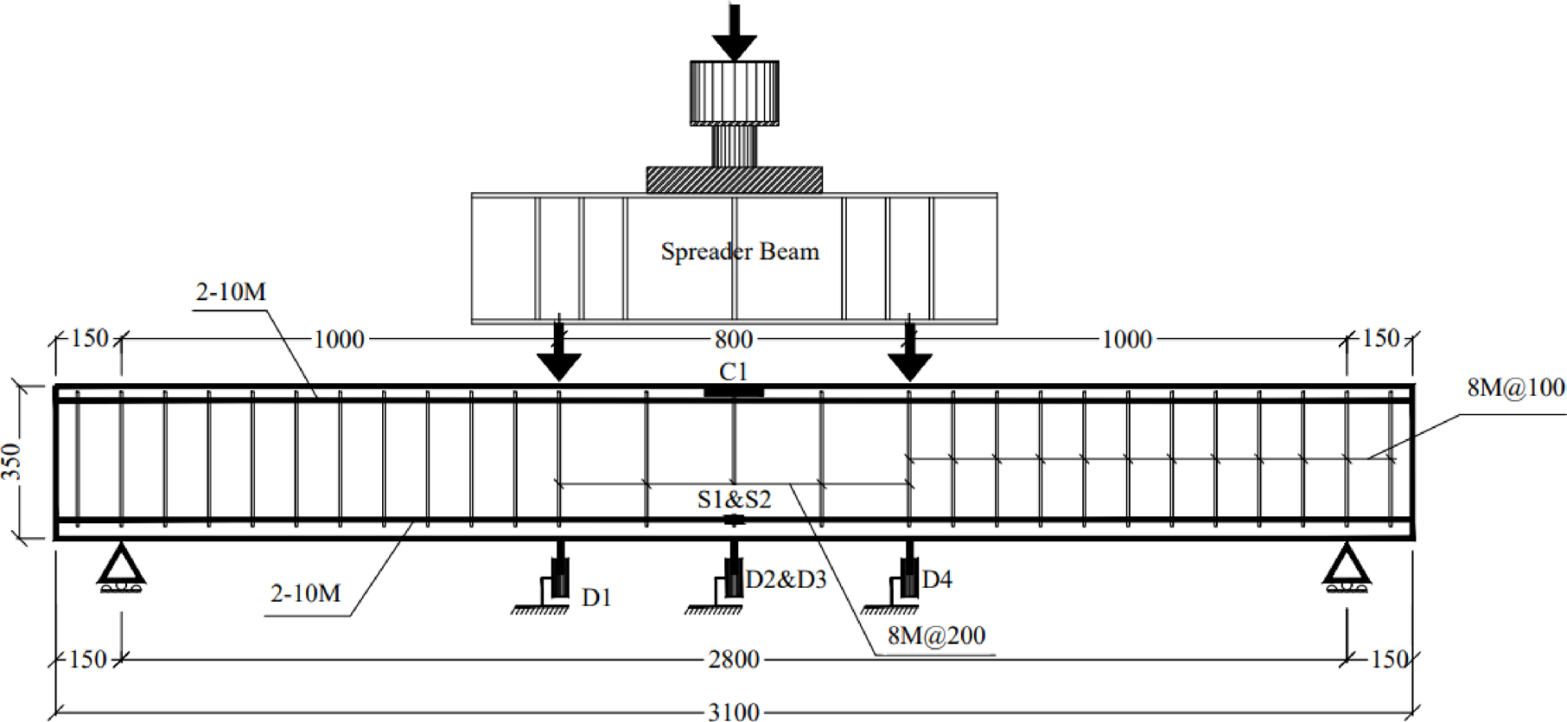
Schematic drawing for instrumentation and test setup.

Details of the instrumentation are shown in Fig. 5. The deflection along the beams’ span was monitored using four linear variable differential transducers (LVDTs) accurate to 0.001 mm, labeled D_1_ to D_4_ (D_2_ & D_3_ at mid-span, and D_1_ & D_4_ under the concentrated loads). Crack propagation was also monitored during testing until failure. Before testing, the beams were painted white to make crack monitoring easier. Moreover, an automatic data-acquisition system (DAQ) connected to a computer was used to monitor loading and deflections.

### Test Setup and Procedure

**Fig. 6:**
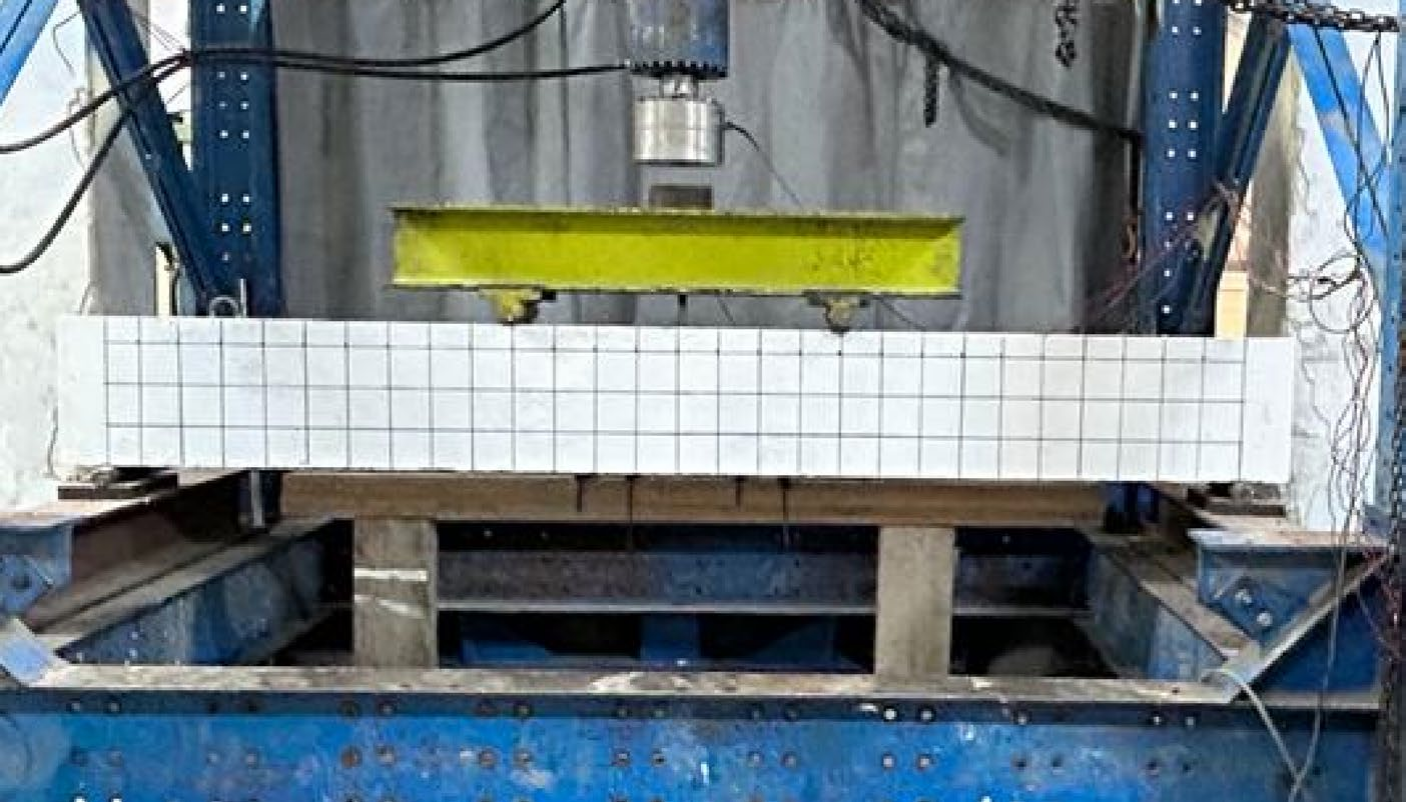
Overview of the test setup.

The eight beams were simply supported and loaded under four-point bending until failure. Figure 5 shows the dimensions and locations of the applied loads, while Fig. 6 provides a photo of the test setup. The beams were visually inspected throughout testing until the first crack appeared, and the corresponding load was recorded. The cracking load was also confirmed based on the change in stiffness of the load–deflection and load–strain relationships.

## Test results and discussion

The beams are divided into three groups in discussion according to the investigated parameters. For each group, the results are revealed and discussed in terms of the crack pattern, load–deflection response, ultimate capacity, and mode of failure. Finally, the ductility indices are discussed for all applied strengthened beams. Group (I) consists of B1-C, B2-S-4L, and B3-S-2L, which discuss the effect of number of BFRP layers used in strengthening and the efficiency of BFRP as a strengthening material. Group (II) consists of beams B1-C, B3-S-2L, B4-0.91S-2L, and B5-0.54S-2L, which discuss the effect of development length required to change the debonding failure mode to BFRP rupture. Group (III) consists of beams B1-C, B4-0.91S-2L, B5-0.54S-2L, B6-0.54S-2L-U, B7-0.54S-2L-SP75, and B8-0.54S-2L-SP150, which discusses the efficiency of anchorage systems (U-wraps and spike anchors) in the enhancement of the ultimate flexural moment of strengthened beams.

It should be noted that some of the investigated parameters in the experimental program are not entirely independent. In particular, the effects of strengthening length, end detailing, and anchorage systems are partially interrelated in certain specimens. Therefore, the observed structural responses in some cases represent the combined influence of these parameters, which is taken into consideration in the interpretation of the results. Therefore, it is recommended that further studies investigate the effect of each parameter individually.

### Deflection response

**Fig. 7:**
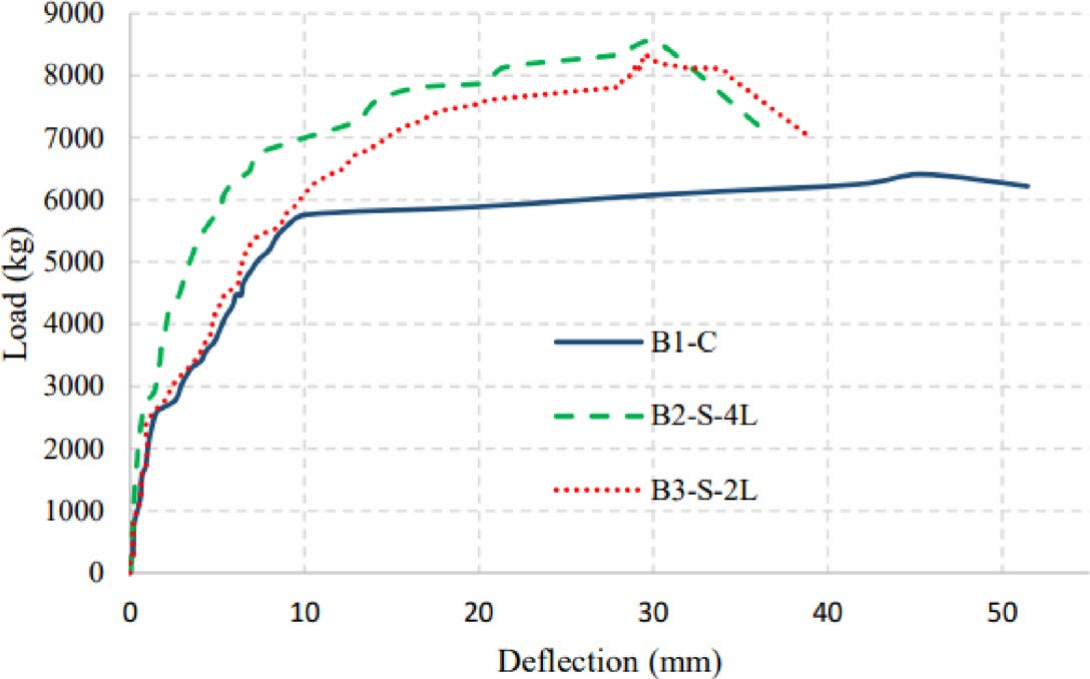
Applied Load–mid-span deflection relationships for group (I).

**Fig. 8:**
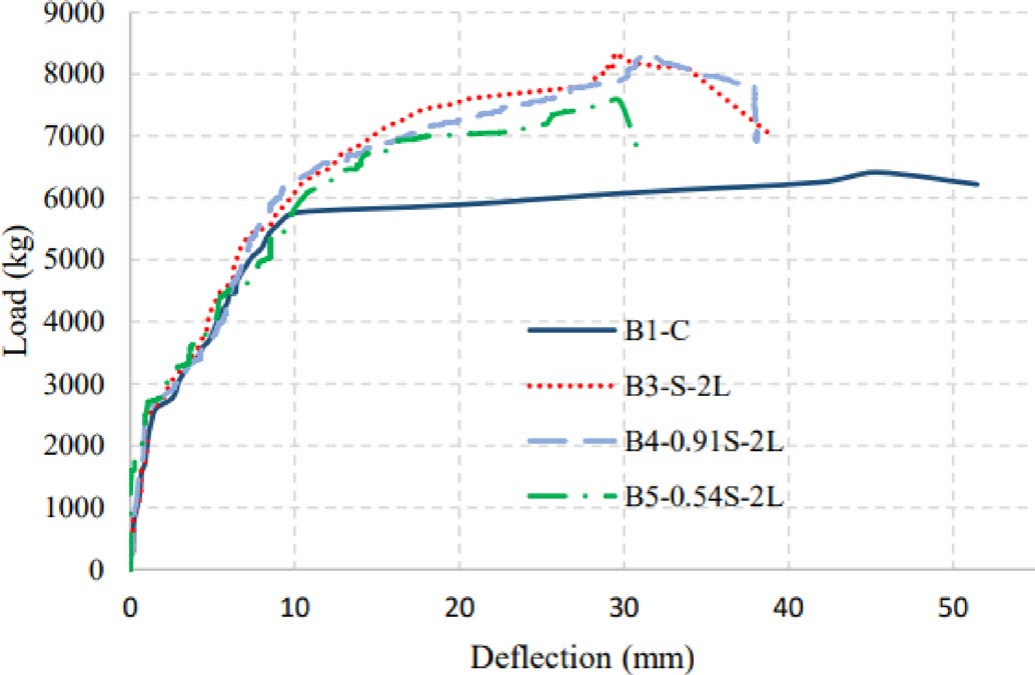
Applied load–mid-span deflection relationships for group (II).

**Fig. 9:**
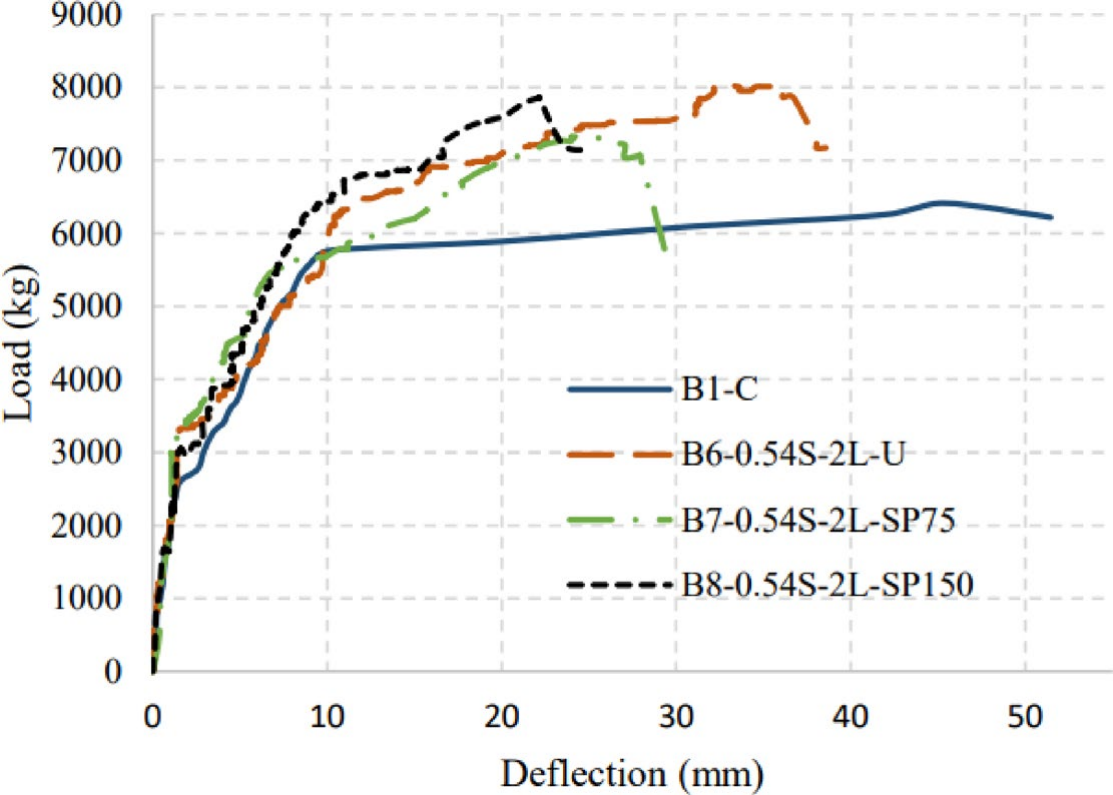
Applied load–mid-span deflection relationships for group (III) beams.

**Fig. 10:**
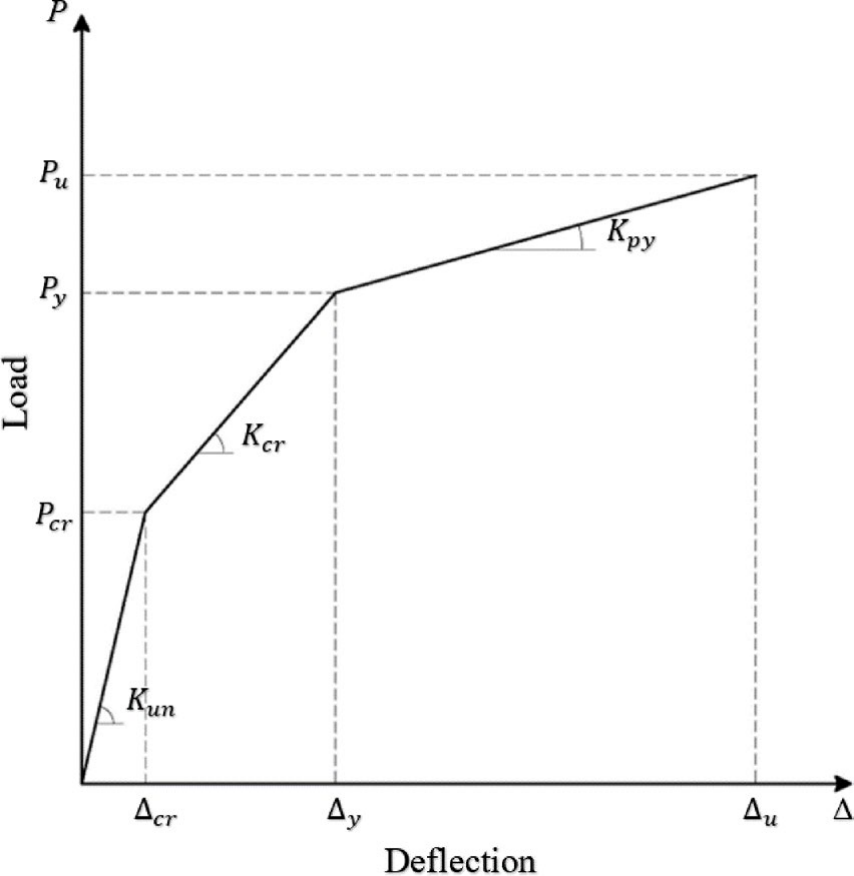
Idealized trilinear load versus mid-span deflection curve.

Figures 7, 8, and 9 show the applied load versus the average mid-span deflection responses at positions D2 and D3 for group (I), group (II), and group (III) beams, respectively. The relationship between load and the average mid-span deflection showed three different responses corresponding to the major changes in section stiffness. In the beginning, the beams behaved linearly with uncracked stiffness up to cracking load. After that, the beam started to resist with its cracked section with degraded stiffness up to yielding of main steel reinforcement. Finally, due to the strain hardening nature of low carbon steel bars the beam showed sharp decrease in its post-yielding stiffness up to failure. An idealized curve representing trilinear behavior of load versus mid-span deflection response is shown in Fig. 10.

In group (I) beams, a very small increase in stiffness appears in the deflection curves in the pre-cracking phase, as the slope of each beam’s load–deflection curve increases with the increase in BFRP layers. Post-cracking, the strengthened beams have a notable increase in stiffness when compared to the control beam, which provides an increase in yield and ultimate capacity of the beam. After yielding of the tension steel, the specimens continued to carry an increasing load until either rupture or debonding of the BFRP occurred.

### Cracking load and pattern

**Fig. 11:**
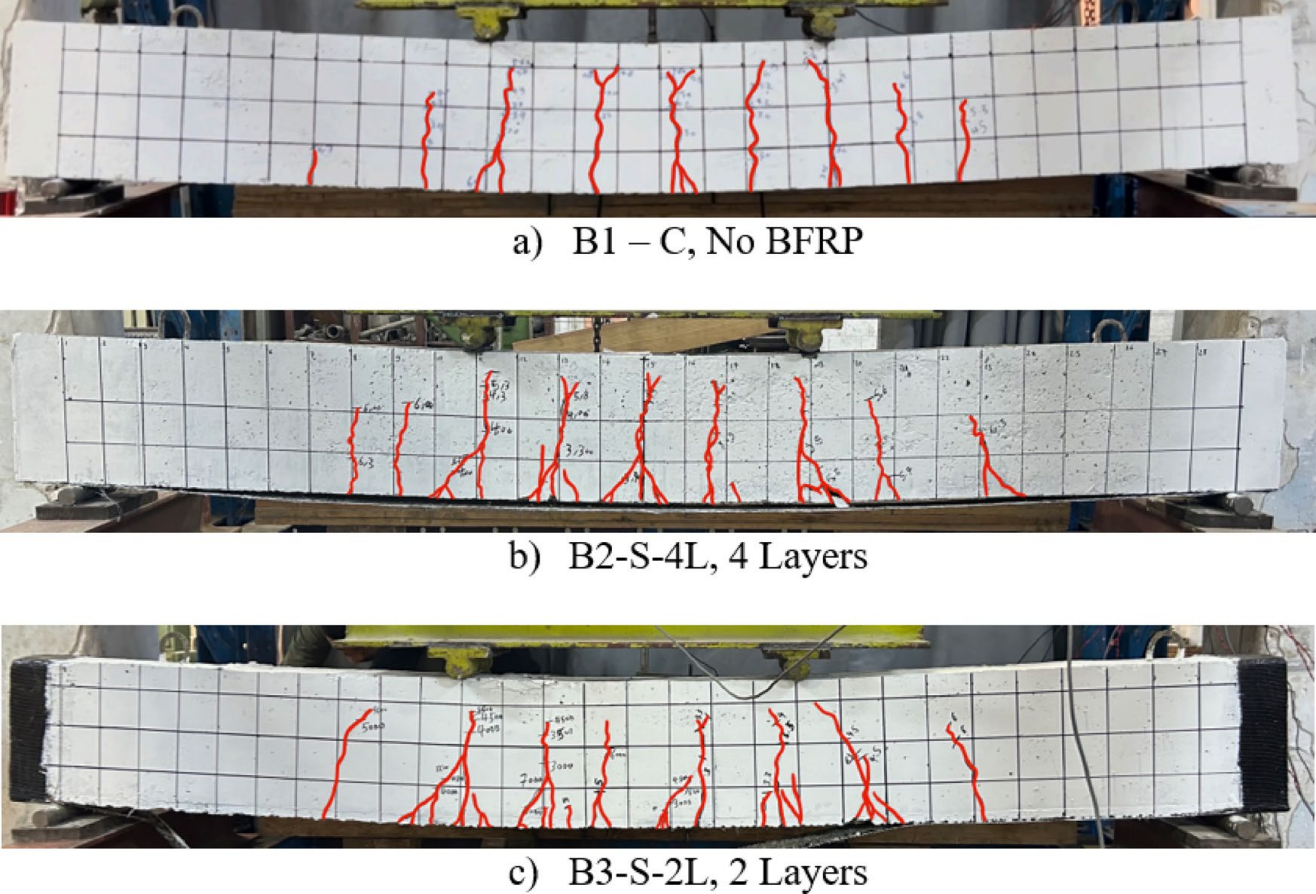
Crack pattern and failure modes of group (I).

The crack propagation in the tested beams followed the flexural cracking patterns often seen in simply supported beams. The formation of cracks started between the two concentrated loads, in the zone of constant flexural moment. The cracks were vertical and perpendicular to the direction of the maximum principle tensile stress induced by pure bending. As the load increased, more flexural cracks appeared so the spacing between cracks decreased. Although direct measurements of crack width were not recorded, it is clear that by increasing the number of BFRP layers, a higher number of flexural tensile cracks emerged as expected which indicates smaller crack widths, as BFRP layers increase the axial stiffness ($E_fA_f$), which enhances stress distribution along the beam and delays crack widening compared to the control beam^26^. However, the lack of quantitative crack width measurements is acknowledged as a limitation of the current study. The crack patterns for Group (I) at the end of the tests are shown in Fig. 11.

The cracking moment of all the tested beams ranged from 24.8 to 31.0 kN m with an average value of 27.8 kN m. Experimental cracking moments of the beams were determined by the load at first crack, or where a remarkable reduction in stiffness was seen in the load–deflection relationship. The cracking moments were also calculated according to Eq. (1). The cracking moments ($M_{cr}$) of each tested beam are shown in Table 4. In addition, Table 4 presented a comparison between the experimental and predicted values of the cracking moments according to ACI 318-19^27^. It was found that the number of layers of BFRP had no significant effect on the cracking moment and the cracking moment is significantly affected by the modulus of rupture of concrete (*f*_*r*_).

1 $$M_{cr} = \frac{{f_{r} \times I_{g} }}{{y_{t} }}$$

where the modulus of rupture of concrete (*f*_*r*_) is calculated from Eq. (2) according to ACI 318-19.

2 $$f_{r} = 0.62\sqrt {f_{c}^{\prime} }$$

**Table 4:** Analytical predictions of failure mode and nominal moment.

Beam ID	Predicted (kN.m) ACI 440.2R-17		
	M_cr_	M_*n*_	MOF ^a^
B1-C	23.6	54.0	Steel yielding
B2-S-4 L	23.6	98.0	Debonding
B3-S-2 L	23.6	88.5	FRP Rupture
B4-0.91 S-2 L	23.6	88.5	FRP Rupture
B5-0.54 S-2 L	23.6	88.5	Debonding
B6-0.54 S-2 L-U	23.6	88.5	FRP Rupture
B7-0.54 S-2 L-SP75	23.6	88.5	Debonding
B8-0.54 S-2 L-SP150	23.6	88.5	Debonding

As shown in Table 4, the average cracking moments of all beams were generally higher than those predicted according to ACI 318-19 by 18%. Bischoff^28^ stated that additional hair cracks can be caused by extra stresses resulting from shrinkage, and temperature effects, which lowers the cracking load.

### Ultimate capacity and modes of failure

Table 4 presents the flexural capacity (*M*_*n*_) for all the tested beams. B1-C beam was designed as an under-reinforced section to fail due to steel yielding like the common design concept in RC beams in real applications. Using Eq. (3), the balanced reinforcement ratios were computed. For ACI 318-19, the terms (*α*_*1*_) and (*β*_*1*_) were computed using Eqs. (4a) and (4b), respectively. B2-S-4L was predicted to fail by BFRP debonding from concrete substrate by calculating the debonding strain using Eq. (5). In B3-S-2L the BFRP layers passed over the support, and a full wrapping scheme was applied at the ends of the beam with 100 mm width to ensure BFRP rupture. The increase in flexure capacity was insignificant between the beams strengthened by two and four layers due to different failure modes.

3 $$\rho_{fb} = \alpha_{1} \beta_{1} \frac{{f_{c}^{\prime} }}{{f_{fu} }}\frac{{\varepsilon_{cu} }}{{\varepsilon_{cu} + \varepsilon_{fu} }}$$

4a $$\alpha_{1} = 0.85$$

4b $$\beta_{1} = 0.85 - 0.05\left( {f_{c}^{\prime} - 27.6} \right)/6.9$$

5 $$\varepsilon_{fd} = 0.41\sqrt {\frac{{f_{c}^{\prime} }}{{nE_{f} t_{f} }} \le 0.9\varepsilon_{fu} }$$

In B4-0.91S-2L, the test parameter of development length required to prevent premature debonding of FRP is investigated. The development length is calculated using Eq. (6) according to (ACI 440.2R-17), so the length of BFRP layers applied was 2570 mm with curtailment of 115 mm from each side. The mode of failure of B4-0.91S-2L was FRP rupture which verifies that when the development length (*L*_*df*_) of BFRP is applied, the failure mode changed from delamination of FRP layers from concrete substrate (as in B5-0.54S-2L) to FRP rupture (as in B4-0.91S-2L).

6 $$l_{df} = \sqrt {\frac{{nE_{f} t_{f} }}{{f_{c}^{\prime} }}}$$

The ultimate capacity of the test specimens was predicted using the strain compatibility approach in ACI 318-19^27^ and compared to the measured values. The analytical predictions of failure mode and nominal moment capacity for all specimens are summarized in Table 4, while Table 5 shows the experimental-to-predicted ultimate capacity of the tested beams which is calculated according to Eqs. (7) and (8). The ACI 318-19 approach yielded an average experimental-to-predicted ultimate capacity of 0.93 ± 0.11. The *β*_*1*_ factor is calculated according to ACI 318-19 and the assumed concrete strain at ultimate load is 0.003.

7 $$f_{f} = \left( {\sqrt {\frac{{\left( {E_{f} \varepsilon_{cu} } \right)^{2} }}{4} + \frac{{0.85\beta_{1} f_{c}^{\prime} }}{{\rho_{f} }}E_{f} \varepsilon_{cu} } - 0.5E_{f} \varepsilon_{cu} } \right) \le f_{fu}$$

8 $$M_{n} = \rho_{f} f_{f} \left( {1 - \frac{{\rho_{f} f_{f} }}{{2\alpha_{1} f_{c}^{\prime} }}} \right)bd^{2}$$

**Table 5:** Cracking, yield and ultimate moments and mode of failure.

Beam ID	Experimental (kN.m)				Capacity Enhancement (%)	Predicted (kN.m) ACI 440.2R-17		*M*_*cr,Exp.*_*/M*_*cr,Pre*_	*M*_*n,Exp.*_*/M*_*n,Pre*_
	*M*_*cr*_	*M*_*y*_	*M*_*n*_	MOF ^a^		*M*_*cr*_	*M*_*n*_		
B1-C	26.1	57.6	64.1	SY + SU	–	23.6	54.0	1.11	1.19
B2-S-4L	29.4	64.6	85.2	D	33	23.6	98.0	1.25	0.87
B3-S-2L	26.7	62.4	81.4	SY + R	27	23.6	88.5	1.13	0.92
B4-0.91S-2L	27.6	62.2	83.0	SY + R	29	23.6	88.5	1.17	0.94
B5-0.54S-2L	26.9	56.1	75.7	SY + D	18	23.6	88.5	1.14	0.86
B6-0.54S-2L-U	31.0	61.0	80.0	SY + R	25	23.6	88.5	1.31	0.90
B7-0.54S-2L-SP75	24.8	55.3	75.4	SY + D	18	23.6	88.5	1.05	0.85
B8-0.54S-2L-SP150	29.7	64.5	78.6	SY + D	23	23.6	88.5	1.26	0.89
	Average				1.18	0.93^c^			
	Standard deviation				0.09	0.11			

^a^SY + SU: Steel yielding followed by steel rupture; D: Delamination of BFRP sheets; SY + R: Steel yielding followed by rupture of BFRP sheets; SY + D: Steel yielding followed by BFRP delamination.

**Fig. 12:**
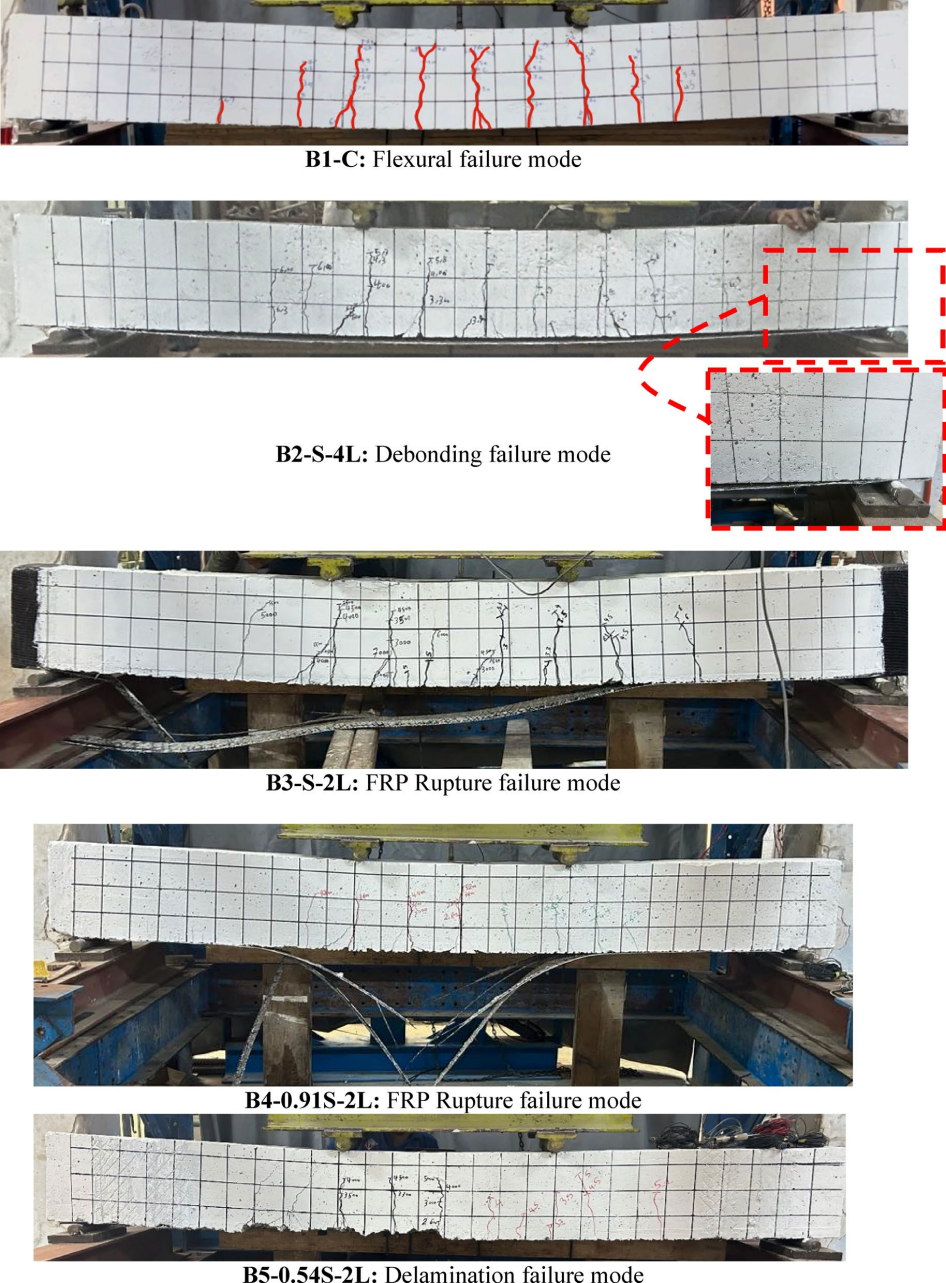
Failure modes of tested beams.

For group (I) beams that include different number of BFRP layers, using four layers of BFRP layers in B2-S-4L, the ultimate capacity of the beams was enhanced by 33% compared to the control beam, in spite of the debonding mode of failure. The four layers of BFRP slipped from the concrete substrate from one side, which means insufficient anchorage by passing the layers under the support. However, B3-S-2L strengthened using two layers of BFRP enhanced the capacity by 27% and the mode of failure was FRP rupture affected by the used U-wraps at the beam ends to avoid premature debonding and ensure full usage of BFRP. The modes of failure of group (I) are shown in Fig. 12. It is worth mentioning that the failure of FRP was adjacent to the support, not in the middle of the beam as expected. This may be attributed to combined stresses concentrations in FRP till failure [Direct shear due stress concentrations over the support and tension stress due to bending moment].

For group (II) beams, different development lengths were used to prevent premature debonding failure mode. Reference to beam B4-0.91S-2L, it was found that the development length calculated from ACI was enough to change the mode of failure from debonding to FRP rupture with full utilization of BFRP strength, in addition to enhancement of flexural capacity by 29% compared to the control beam. Consequently, to evaluate the efficiency of different anchorage systems, two layers of BFRP are used in B5-0.54S-2L with length 1500 mm, which represents only 54% of the span. This length covers 800 mm of constant moment zone and extends 350 mm each side. The mode of failure was concrete cover delamination as expected with 18% enhancement in capacity of the beam compared to the control beam. Modes of failure for beams B4-0.91S-2L and B5-0.54S-2L are shown in Fig. 12.

For group (III) beams, different anchorage systems (U-wrapp and Spike anchors) were used and investigated. To examine the efficiency of using U-wrap as an anchorage system, B6-0.54S-2L-U was prepared and tested. B6-0.54S-2L-U failed in flexure at 80 kNm which represented a significant flexural strength gain of 25% compared to the control specimen. The mode of failure in B6-0.54S-2L-U was FRP rupture with full usage of BFRP strength. The strengthened beams B7-0.54S-2L-SP75, and B8-0.54S-2L-SP150 enhanced using spike anchors made using BFRP of 75 mm and 150 mm embedded depth, respectively. B7-0.54S-2L-SP75 failed by concrete cover delamination at an ultimate load approximately equivalent to B5-0.54S-2L which is without any anchorage system which proved the weakness of the spike anchors. In addition, beam B8-0.54S-2L-SP150 failed in flexure at 78.6 kNm with 23% enhancement compared to the control beam. However, B8-0.54S-2L-SP150 failure mode was FRP delamination.

A more detailed comparison between experimental and predicted results reveals that the accuracy of the design provisions is strongly influenced by the failure mode and bond behavior of the BFRP system. For specimens that failed by premature debonding, such as B2-S-4L and B5-0.54S-2L, the predictions based on ACI 440.2R-17 tended to overestimate the flexural capacity. This can be attributed to the simplified assumptions regarding effective FRP strain and ideal bond conditions, which do not fully capture the complex interfacial behavior leading to early debonding.

On the other hand, for specimens where adequate anchorage or sufficient development length was provided, such as B3-S-2L, B4-0.91S-2L, and B6-0.54S-2L-U, the failure mode shifted to BFRP rupture, resulting in better agreement between experimental and predicted values. This indicates that when full composite action is achieved, the design provisions provide more reliable estimates of flexural capacity.

Furthermore, the comparison highlights that ACI 318-19, when used in conjunction with simplified assumptions, generally provides conservative estimates of the flexural capacity. However, it does not explicitly account for the contribution of externally bonded FRP systems, which limits its applicability for strengthened members. The observed discrepancies emphasize the importance of considering anchorage efficiency, bond characteristics, and failure mechanisms when applying design guidelines. These factors play a critical role in determining the effective utilization of BFRP and the accuracy of analytical predictions.

To evaluate the contribution of anchorage systems, an anchorage effectiveness factor (*k*_*fab*_) is defined as the ratio between the ultimate flexural capacity of the anchored specimen and that of the corresponding unanchored specimen with the same strengthening configuration. This parameter provides a quantitative measure of the efficiency of the anchorage system in enhancing load transfer and preventing premature debonding.

Higher values of *k*_*fab*_ indicate improved utilization of the BFRP material and a more effective anchorage system. However, it should be noted that the calculated values are based on a limited number of specimens and are intended for comparative purposes within this study rather than as generalized design parameters.

### Ductility of the tested beams

Ductility is a significant measure for RC beams subjected to flexural loading as it describes the amount of energy absorbed prior to failure^29^. The deflection ductility index is calculated for all beams using Eq. (9) which is utilized in previous studies^30,31^. The displacement ductility index (μ_Δ_) was calculated as the ratio between the ultimate deflection (Δ_u_) and the deflection at steel yielding (Δ_y_). The yield point was determined from the load–deflection curve using the deviation from linearity, while the ultimate deflection corresponds to the maximum recorded load. This definition is consistent with previous studies on FRP-strengthened RC beams.

9 $$\mu_{\Delta } = \frac{{\mu_{u} }}{{\mu_{y} }}$$

Table 6 shows the values of ductility indices of all tested beams and the percentage decrease of beams for which the mode of failure was BFRP rupture. It was found that the strengthened RC beams had lower ductility values than the beams without strengthening while the percentage decrease limited to 29% which matches with Duic et al.^21^. Using four layers of BFRP didn’t decrease the ductility of the beam from the control beam which may be due to the debonding failure mode or due to optimum number of layers used as stated by Stephen et al.^32^, although using two layers of BFRP reduced the ductility by 27% with BFRP rupture failure mode. Using U-wraps in B6-0.54S-2L-U as an anchorage system enhances the ductility of the strengthened beam over B4-0.91S-2L by 2%.

**Table 6:** Ductility indices of tested beams.

No	Spec. Code	Δ_y_	Δ_u_	Ductility	Ductility decrease
1	B1-C	10.09	45.33	4.5	–
2	B2-S-4L	6.83	30.33	4.4	–
3	B3-S-2L	9.57	31.50	3.3	27
4	B4-0.91S-2L	9.53	31.10	3.3	27
5	B5-0.54S-2L	9.47	29.75	3.1	–
6	B6-0.54S-2L-U	10.40	33.60	3.2	29
7	B7-0.54S-2L-SP75	7.85	24.86	3.2	–
8	B8-0.54S-2L-SP150	10.26	20.14	1.9	–

## Summary and conclusion

This paper presented an experimental study aimed at investigating the flexural behavior of concrete beams strengthened with basalt-fiber-reinforced-polymer sheets (BFRP). A total of eight concrete beams were constructed and tested up to failure. The beams were tested under four-point bending over a clear span of 2800 mm until failure, and the experimental results were compared to design provisions. The test parameters were the number of BFRP layers, the development length and the effectiveness of U-wrap and spike anchorage systems. Based on the test results and the discussions presented herein, the following conclusions can be drawn:

1. BFRP composite is a green and efficient material to be used for flexural strengthening of RC beams. The load-carrying capacities for beam strengthened with two layers of BFRP sheets [B4-0.91S-2L] was increased by 29% compared to the control beam.
2. As the amount of BFRP sheets increases for flexure strengthening, the usage efficiency of BFRP will be decreased due to premature debonding. Using two layers of BFRP enhanced the flexural capacity by 27% [B3-S-2L] with BFRP rupture failure mode, while doubling the number of layers [B2-S-4L] enhanced the flexural capacity by 33% only, due to premature debonding failure mode. From a practical design perspective, increasing the number of BFRP layers without providing adequate anchorage is not recommended, as it may lead to premature debonding and inefficient utilization of the strengthening material. Proper anchorage systems are therefore essential to ensure that the full tensile capacity of BFRP is mobilized.
3. Development length calculated from ACI 440.2R-17 has been verified to prevent premature debonding of BFRP layers and FRP rupture occurs [B4-0.91S-2L].
4. Using BFRP sheets for strengthening of RC beams decreases the ductility by an average value of 28% when compared to the ductility of the control beam.
5. The anchorage systems affected the failure mode of the strengthened beams. Failure modes are changed from plate end delamination to FRP rupture using U-wraps, while spike anchors didn’t change the failure mode but enhanced the ultimate capacity of the beams.
6. Using U-wraps as an anchorage system increased the beam strength by 25% compared to the control beam, and the beams failed due to BFRP rupture.
7. Spike anchors of 150 mm embedded depth enhanced the strength of the beam by 23% [B8-0.54S-2L-SP150] compared to the control beam, which is slightly less effective than using U-wraps (B6-0.54S-2L-U). However, reducing the embedded depth to 75 mm was ineffective [B7-0.54S-2L-SP75], as it enhanced the beam capacity by 18% only compared to the control beam which is similar to beam (B5-0.54S-2L) without anchorage systems.
8. U-wraps have anchorage effectiveness factor *k*_*fab*_ = 2.36, while spike anchors have anchorage effectiveness factor *k*_*fab*_ = 1.97.
9. Generally, U-wraps have higher efficiency than spike anchors. In addition to that U- wraps are easy to install and non-destructive, which makes them an ideal choice for beam strengthening applications.

## Future studies and recommendations

Future research is recommended to investigate the long-term durability of BFRP anchorage systems, including environmental effects and fatigue loading. Further studies are also needed to evaluate different anchorage configurations and to develop analytical models for predicting anchorage efficiency in FRP-strengthened RC members.

## Data Availability

The datasets generated and/or analysed during the current study are available from the corresponding author on reasonable request.
